# Joint patient and clinician priority setting to identify 10 key research questions regarding the long-term sequelae of COVID-19

**DOI:** 10.1136/thoraxjnl-2021-218582

**Published:** 2022-03-30

**Authors:** Linzy Houchen-Wolloff, Krisnah Poinasamy, Kate Holmes, Maryrose Tarpey, Claire Hastie, Kelly Raihani, Natalie Rogers, Nikki Smith, Dawn Adams, Paul Burgess, Jean Clark, Clare Cranage, Mahadev Desai, Nicola Geary, Rhyan Gill, Jitendra Mangwani, Lily Staunton, Colin Berry, Charlotte E Bolton, Trudie Chalder, James Chalmers, Anthony De Soyza, Omer Elneima, John Geddes, Simon Heller, Ling-Pei Ho, Joseph Jacob, Hamish McAuley, Aarti Parmar, Jennifer K Quint, Betty Raman, Matthew Rowland, Amisha Singapuri, Sally J Singh, David Thomas, Mark R Toshner, Louise V Wain, Alex Robert Horsley, Michael Marks, Christopher E Brightling, Rachael A Evans

**Affiliations:** 1 Respiratory Sciences, University of Leicester, Leicester, UK; 2 NIHR Leicester Biomedical Research Centre - Respiratory, University of Leicester, Leicester, UK; 3 Research and Innovation Advocacy, Asthma & Lung UK, London, UK; 4 Office for Clinical Research Infrastructure (NOCRI), National Institute for Health Research, London, UK; 5 James Lind Alliance, University of Southampton, Southampton, UK; 6 Long Covid Support, NIHR Leicester Biomedical Research Centre - Respiratory, Leicester, UK; 7 Patient and Public Involvement Group, NIHR Leicester Biomedical Research Centre - Respiratory, Leicester, UK; 8 Leicester Orthopaedic Research Network, University Hospitals of Leicester NHS Trust, Leicester, UK; 9 British Heart Foundation Glasgow Cardiovascular Research Centre, University of Glasgow, Glasgow, UK; 10 Respiratory Medicine, NIHR Nottingham Biomedical Research Centre Respiratory Theme, University of Nottingham, Nottingham, UK; 11 Pyschological Medicine, King's College London, London, UK; 12 Molecular and Clinical Medicine, School of Medicine, University of Dundee, Dundee, UK; 13 Lung Biology and Transplantation Group, Newcastle University, Newcastle upon Tyne, UK; 14 Department of Psychiatry, University of Oxford, Oxford, UK; 15 Department of Oncology and Metabolism, The University of Sheffield, Sheffield, UK; 16 Medical Research Council Human Immunology Unit, Weatherall Institute of Molecular Medicine, Oxford, UK; 17 Oxford Centre for Respiratory Medicine, Churchill Hospital, Oxford, UK; 18 Centre for Medical Imaging and Computing, University College London, London, UK; 19 Department of Respiratory Medicine, University College London, London, UK; 20 Respiratory Epidemiology, Occupational Medicine and Public Health, Imperial College London, London, UK; 21 Division of Cardiovascular Medicine, Radcliffe Department of Medicine, University of Oxford, Oxford, UK; 22 Nuffield Department of Clinical Neurosciences, University of Oxford, Oxford, UK; 23 Department of Immunology and Inflammation, Imperial College London, London, UK; 24 Pulmonary Vascular Disease Unit, Papworth Hospital NHS Foundation Trust, Cambridge, UK; 25 School of Clinical Medicine, University of Cambridge, Cambridge, UK; 26 Genetic Epidemiology Group, Department of Health Sciences, University of Leicester, Leicester, UK; 27 Respiratory Medicine, Manchester University NHS Foundation Trust, Manchester, UK; 28 Department of Clinical Research, London School of Hygiene & Tropical Medicine, London, UK

**Keywords:** COVID-19

## Abstract

Given the large numbers of people infected and high rates of ongoing morbidity, research is clearly required to address the needs of adult survivors of COVID-19 living with ongoing symptoms (long COVID). To help direct resource and research efforts, we completed a research prioritisation process incorporating views from adults with ongoing symptoms of COVID-19, carers, clinicians and clinical researchers. The final top 10 research questions were agreed at an independently mediated workshop and included: identifying underlying mechanisms of long COVID, establishing diagnostic tools, understanding trajectory of recovery and evaluating the role of interventions both during the acute and persistent phases of the illness.

## Introduction

Since its first description 2 years ago to date, the SARS-CoV-2 has infected at least 250 million people worldwide and resulted in over 5 million deaths.^1^ For survivors, there is a high rate of delayed recovery, ongoing symptoms, reduced health-related quality of life and inability to return to work.^2^ ‘long COVID’ describes the persistence of symptoms or disability after the acute infection, not explained by an alternative diagnosis.^3^ In patients hospitalised with COVID-19, only 3 out of 10 patients felt fully recovered at 6 months^4^ and 12 months postdischarge.^5^ With over half a million adults admitted to hospital in the UK to date,^6^ symptomatic survivors of COVID-19, represent a large and growing population.

Given the persistence of the coronavirus pandemic and the large numbers of people affected, it is important to define research priorities to aid effective targeting of resources. Previous attempts to do this have focused on the research priorities of the clinical community in adults^7^ and airways disease.^8^ The WHO and International Severe Acute Respiratory and emerging Infection Consortium have recently published six key research priorities for coronavirus which were refined through a multistakeholder forum.^9^ Research prioritisation involves a broad reach of patient and clinical stakeholders as well as considering questions of feasibility. One approach successfully deployed in other disease areas is that of the James Lind Alliance (JLA), a non-profit-making initiative partly funded by the National Institute for Health Research. The JLA has a well-established process to ensure that those most affected by a condition are involved in prioritising research (https://www.jla.nihr.ac.uk/).

The post-hospital COVID-19 (PHOSP-COVID) study is a UK-wide national research collaboration examining the long-term sequelae of COVID-19 (https://www.phosp.org). Over 7500 patients discharged from over 80 UK hospitals between March 2020 and March 2021 were recruited by March 2022. This places the PHOSP-COVID consortium in a unique position to establish a priority setting partnership (PSP) for research into long COVID. The aim was to produce a top 10 research priority question list for survivors of a hospital admission with COVID-19.

## Methods

**Figure 1:**
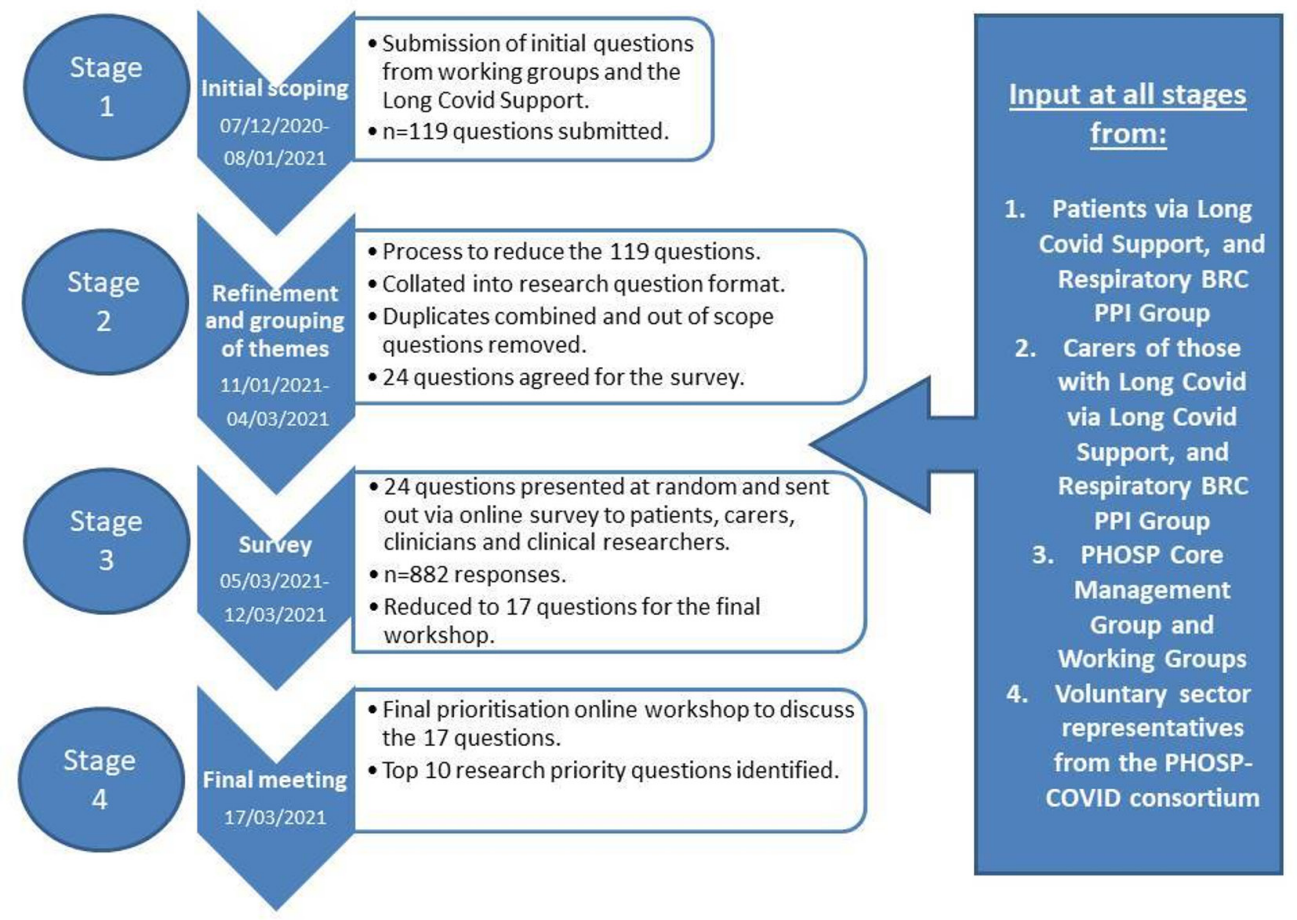
Methodology of the research prioritisation process and input at all stages from key stakeholders. BRC PPI, Biomedical Research Centre Patient and Public Involvement; PHOSP, post-hospitalisation COVID-19.

Our PSP took place between December 2020 and March 2021 and incorporated views from adults with self-reported experience of long COVID (both hospitalised and non-hospitalised for the acute illness), carers, clinicians and clinical researchers. We used an adapted version of the JLA process^10^ as outlined in figure 1. The elicitation survey requested contributors answer the following: ‘what questions would you like to see answered by research into the longer term consequences post-hospital admission for COVID-19?’ This was shared with multiple patients, clinicians and relevant stakeholders including members of the 13 PHOSP-COVID working groups (step 1) detailed in online supplemental figure S1. The initial questions were combined, reworded (step 2) and then shared across multiple platforms via an online prioritisation survey (step 3). Survey questions were presented in random order for each individual. The final top 10 research questions were agreed at a dedicated prioritisation workshop (online supplemental figure S2) mediated by independent JLA facilitators and hosted via videoconference (step 4). Ethical approval was not required but patients and clinicians provided verbal consent to be recorded during the workshop.

10.1136/thoraxjnl-2021-218582.supp1 Supplementary data

## Results

The elicitation survey generated 119 questions from long Covid Support (patient group) and the PHOSP-COVID working groups (online supplemental tables S1 and S2). There was considerable overlap in questions posed by patients and clinicians and between different working groups. Similar questions around specific organs/symptoms were combined and questions outside of scope were omitted. A refined list of 24 questions was reviewed by patient groups to ensure wording was clear (online supplemental table S3).

There were 882 respondents to the online survey of whom 819 (93%) were individuals with self-reported long COVID. There was consistency in the prioritisation shown between individuals with self -reported long COVID who were either hospitalised or non-hospitalised (online supplemental table S4). The highest ranked 17 questions were selected from this for the prioritisation workshop (online supplemental table S5). The final list of top 10 priority questions is shown in box 1.

Box 1 **Outcome of research prioritisation: final list of top 10 research questions (not ranked).**

1. What are the underlying mechanisms of long COVID that drive symptoms and/or organ impairment?
2. What imaging techniques or scans may be able to detect and predict the development of organ problems or wider systemic issues?
3. What happens to the immune system throughout patients’ recovery from COVID-19?
4. What can data at 6 and 12 months tell us about the long-term trajectory of illness?
5. What blood or other laboratory tests may be able to detect and predict the development of organ problems or wider systemic issues?
6. What is the impact of treatment(s) during the acute (initial) stage of COVID-19 on recovery?
7. What are the problems within the muscles associated with symptoms limiting activity/function/exercise? If so, what can be done to help?
8. What medications, dietary changes, supplements, rehabilitation and therapies aid recovery?
9. What can be done to support mental well-being during recovery?
10. What is the risk of future adverse health events (eg, stroke, heart attack)?

## Discussion

In this codeveloped priority setting process, we have identified key research priorities for improving our understanding of long COVID. Patients, patient charities and carers were closely involved throughout the process, including in question generation, phrasing and prioritisation. The final research question list was, therefore, broad and reflects the major problems reported 1-year postdischarge from hospital.^2 5^ It differs from previous reports published earlier in the pandemic, which were largely informed by clinician input.^7 8^ Our identified priorities included understanding the underlying mechanisms of long COVID, which drive ongoing symptomatic illness. Related to this, there was an emphasis on identifying diagnostic and prognostic tools, including both imaging and biomarker-based approaches. Finally, there was also a strong emphasis on the potential role of both pharmacological and non-pharmacological interventions to treat symptoms.

Adherence to the principles of JLA methodology was a strength of this PSP, but the need to generate these data rapidly during a pandemic meant that we were unable to follow the full JLA process. For example, the time scale for delivery was significantly shortened. Shorter response times may have impacted on survey responses. We cannot calculate the response rate as the survey was made available publically and the denominator is, therefore, unknown. We were unable to collect detailed demographic data from the survey respondents, so we cannot be certain how representative the sample is. The survey respondents were biased towards individuals with self-reported long COVID who had not been admitted to hospital. The workshop, however, involved an equal number of hospitalised and non-hospitalised patient attendees, and between those with lived experience or other expertise. As our understanding of long COVID evolves, it may be necessary to re-evaluate research priorities

To summarise, we have completed a comprehensive and inclusive research prioritisation exercise to identify the top priority questions for research to improve outcomes for survivors of a hospital admission for COVID-19. The relevance may extend to people with long COVID who were not hospitalised. Given the large numbers of people with long COVID, and the persistence of the pandemic, this is an important resource to help inform future research strategies and policy.
