# Supplementary material for: Joint patient and clinician priority setting to identify 10 key research questions regarding the long-term sequelae of COVID-19
Source: Thorax. 2022 Mar 30;77(7):717–20. doi: 10.1136/thoraxjnl-2021-218582 (PMC9209667; doi:10.1136/thoraxjnl-2021-218582)
Supplement: Supplementary data [file thoraxjnl-2021-218582supp001.pdf]

Online supplement

Detailed methods

The joint research prioritisation outlined in Figure 1 was undertaken in four stages and followed JLA principles as far as possible. Changes were made to incorporate the practicalities of using online systems rather than face to face meetings, and for rapid results. Question setting and prioritisation involved four groups of contributors, all of whom provided initial questions and input on wording and choice of the final priority list:

1) Adults over the age of 18 years old with lived experience of Long Covid, both community-managed and hospitalised.

2) Carers of adults with lived experience of Long Covid.

3) Clinical researchers from the PHOSP-COVID consortium, drawn from the Core Management Group (CMG) as well as the 13 specialist Working Groups and platforms.

4) Voluntary sector representatives from the PHOSP-COVID consortium.

Patients with lived experience of Long Covid were either part of Long Covid Support, a charitable organisation focused on peer support, advocacy, and research involvement, whose online community has >40,000 members, or the Leicester NIHR Biomedical Research Centre (BRC) Patient and Public Involvement (PPI) group for COVID-19 (predominantly patients post-hospitalisation). The input of these contributors at all stages of the process are summarised in figure 1.

Stage 1: Sourcing questions from individuals with Long Covid and from relevant clinical researchers/clinicians

An initial list of research questions was generated by inviting contributions from all four stakeholder groups. The elicitation survey requested that contributors answer the question “what questions would you like to see answered by research into Long Covid?” Contributions from the PHOSP-COVID consortium were managed by the working groups which were each asked to determine the specific needs and priorities within their specialty and distil these into a specialty-specific list (maximum of 10 distinct questions). A list of the working groups with links to relevant patient charities are shown in figure S1. Different

Houchen-Wolloff L, *et al. Thorax* 2022; 77:717–720. doi: 10.1136/thoraxjnl-2021-218582

specialties had different levels of community engagement to draw on. The Brain working group for example (incorporating a mental health, neurology and cognition focus) was able to draw on previously published patient prioritisation exercises for mental health (10), whilst the Airways working group had conducted a priority setting exercise for their specialty, predominantly clinician-driven (11). The deadline for questions to be submitted was 8th January 2021.

Stage 2: Synthesising the proposed research questions and revision of the questions for accessibility of the language by individuals with Long Covid

The proposed research questions from both patient groups and PHOSP-COVID working groups were reviewed to ensure they met the scope of the PSP. Examples of questions that were out of scope included those that related solely to patients who had not been hospitalised and questions relating to immune responses to vaccination. Where appropriate, organ-specific questions were combined into broader questions addressing the same issue across different organ systems. This refined list of questions then underwent two phases of patient review: firstly by members of the Leicester COVID-19 PPI group, who improved the wording of questions to ensure they could be easily understood. Secondly, the questions were reviewed by members of Long Covid Support to ensure that the intention of the original questions had been retained and that the wording was clear.

Stage 3: Survey of priority questions to identify top 10 patient and top 10 clinician priorities

The list of questions (in lay language), were then reviewed by patients, carers, clinicians and academics through an online survey. This was distributed by email to PHOSP-COVID working group members and circulated for wider review by patients and carers via Twitter and Facebook (Long Covid Support). Respondents were presented with the refined list of priority research questions in a random order and asked to select their personal top 10 priority questions. The online survey was open for one week from 5th March 2021.

Stage 4: Workshop to determine top 10 priority research questions

Finally, an online prioritisation workshop was held to achieve consensus on a top 10 question list. This was held in March 2021 with support from independent advisors from the JLA. Workshop attendees included members of all four stakeholder groups. Prior to the workshop all participants were sent packs containing pre-work materials with the following brief: "The priority questions we are focusing on are specifically to inform the PHOSP team and working groups to allocate further resource to research. This is not a comprehensive list of all possible questions on Long Covid as some cannot be answered by the PHOSP study". Participants were asked to review and familiarise themselves with the list of research questions with instructions to rank the full list of questions in order of priority in which they were asked to review and familiarise themselves with the list of research questions with instructions to rank the full list of questions in order of priority. The workshop process is outlined in figure S2. The workshop opened with a review of the emerging data from the PHOSP-COVID consortium on prevalence of different symptoms at 5 months post infection. This was to ensure that the final prioritisation was able to incorporate evolving learning about the clinical impact of different symptom clusters. Attendees were divided into four breakout groups, each led by a facilitator from the JLA, and containing a mix of clinicians and patients. The first of three breakout sessions was a discussion of the priorities that had been brought to the workshop following the survey, to enable questions and ensure a shared understanding of the priorities across the group. Individuals were asked to identify their top and bottom three questions from the list. In a second breakout session, questions were then defined as high priority if they had been selected as a top priority by at least one participant, but had not been selected as a bottom priority by any participants. Lower priority questions were defined as being a bottom priority for at least one participant, and not a top priority for any participant. All other combinations resulted in questions being identified as medium priority. The results of the first prioritisation breakout session were amalgamated across the four sub-groups by the JLA advisors to reach a first proposal of the Top 10 priorities. Following this, participants were divided into four different sub-groups for a third breakout prioritisation session to review the proposed top 10 and consider whether questions should be re-categorised so that all questions were characterised as either higher or lower priority. A concluding plenary discussion focused on questions that were not consistently high or low

priority across the groups, and a final vote used to reach consensus on the top 10 research questions.

Results

The process was completed over four months from December 2020 to March 2021.

Stage 1: Sourcing questions from PHOSP-COVID Working Groups and individuals with LongCovid

The initial list of possible questions contained 119 separate questions. Thirty four (29%) of these were submitted by patients through Long Covid Support (online supplement Table S1). The remaining 85 questions were proposed by the 18 different Working Groups (online supplement Table S2).

Stage 2: Synthesising and revising the proposed research questions for an online survey

There was considerable overlap in the objectives of research questions posed by patients and clinicians, and between different Working Groups. The initial list of 119 questions was synthesised into broader questions. This was achieved by, for example, collating questions relating to pathophysiology or epidemiology, or combining similar questions around specific organs or symptoms. Questions outside of the scope of the PSP were omitted. Table S3 lists the questions after review by the CMG and rewording by the patient groups with the rationale. The final list of questions for the survey contained 24 questions.

Stage 3: Results of the online survey prioritising research questions

There were 882 respondents to the survey. 819 (93%) were individuals with Long Covid, of whom 758 (93%) had been community managed for their acute illness. 37 (4%) of respondents were carers of individuals with Long Covid and 26 (3%) were clinicians. 15 out of 24 questions were selected as a top 10 priority by either patients or clinicians and were therefore automatically included in the priority setting workshop. Two additional questions were taken forwards – these being the questions ranked number 11 in either patient or clinician groups. There was consistency in the prioritisation shown between individuals with

Houchen-Wolloff L, *et al. Thorax* 2022; 77:717–720. doi: 10.1136/thoraxjnl-2021-218582

Long Covid who were either community managed or hospitalised (Table 1). This left 17 questions taken to the final workshop (Table 2).

Workshop to determine top 10 priority research questions

The workshop was attended by 12 people with Long Covid, of whom six had been hospitalised, four from Long Covid Support and therefore able to draw on the lived experiences of their members as well as themselves, one carer of an adult hospitalised with COVID and 13 clinicians/ clinical researchers involved in the working groups of the PHOSP-COVID study. Five other members of the PHOSP-COVID CMG attended as observers but did not take part in the discussion and five JLA staff attended to facilitate the workshop.

Following the two prioritisation sessions, there was agreement across groups in identifying eight higher priority and six lower priority questions, but there was an inconsistent response for three questions. A final plenary discussion focused on these three questions with an anonymous final online vote taken to establish which two of the remaining questions should be selected (outlined in figure S2). The final list of top 10 research priority questions from the prioritisation exercise is shown in Table 3 (these are not ranked).

Table S1. 34 questions submitted by Long Covid Support. Contains results for process stage 1.

**1.**	**Overarching theme: What causes Long Covid? (Persistent virus, aberrant immune response/inflammation, autoimmunity, other).**
2.	What can be done to aid recovery? (e.g. therapeutics, diet, supplements, exercise/physiotherapy; see Recovery & Treatment section).
3.	What could be done to prevent others developing Long Covid (e.g. early intervention)?
4.	How many people are affected by Long Covid (proportion and absolute numbers), and what are the risk factors? How does this vary by factors including age (including children), sex, ethnicity, socioeconomic group?
5.	Will people recover from Long Covid? How long might this take? Might some people never recover?
6.	Are there different sub-groups within Long Covid?
7.	Are people with Long Covid at risk of developing associated health problems (in the short, medium and/or longer term)?

8. What is the significance of viral persistence (gastrointestinal tract, stool, saliva) in terms of infectiousness?
9. What similarities/differences are there in Long Covid symptoms between hospitalised/non-hospitalised patients, aside from specific post-intensive care/ post-hospitalisation effects?
**Immunity**
**10. Overarching theme: How long does immunity last, with or without the vaccine?**
11. Can the virus hide in the body and reactivate?
12. How safe is the COVID-19 vaccine for different cohorts, e.g. people with Long Covid, children?
**Symptoms**
**13. Overarching theme: What causes the individual symptoms of Long Covid?**
14. Why do symptoms fluctuate or relapse and remit?
15. Why do pronounced symptoms develop weeks or months after infection?
16. Why do tests and scans often show as normal when symptoms seem so pronounced? Is there a need for new diagnostic tests, such as the Oxford University Xenon magnetic resonance imaging (MRI) of the lungs? How can endothelial dysfunction be detected?
17. At what point should a patient seek medical help for symptoms (individually and in combination)?
18. Is the organ damage detected in studies such as Coverscan likely to be temporary?
19. Can Covid cause other illnesses or syndromes such as postural tachycardia syndrome (PoTS), diabetes, autoimmune conditions, MAST cell activation syndrome, myocarditis/pericarditis? How permanent are these likely to be?
20. How does Covid affect the endocrine system? Why do many people experience chronic issues with their sympathetic nervous system, to what extent might this cause other symptoms, and what can be done about this?
21. Why do many people experience changes to their menstrual cycle and/or an exacerbation of Long Covid symptoms in relation to the menstrual cycle?
**Recovery and Treatment**
**22. Overarching theme: What is the role of rest vs exercise in recovery?**
23. What supplements or dietary factors might aid recovery?
24. Is there a role for treatments such as antiviral medication, steroids, immunomodulation, monoclonal antibodies against SARS-CoV-2, anticoagulation, antiplatelets, antihistamines, statins, Angiotensin-converting enzyme (ACE) inhibitors or Angiotensin receptor blockers, for example?
25. How can Covid Clinics best help patients? What scans and tests are useful? What can be done to identify potentially dangerous symptoms before rehab is recommended?
**Respiratory**
**26. Overarching theme: What is the underlying reason for ongoing breathing problems? Do these relate to the lungs, heart or nervous system? What tests might help to identify this?**
27. Why do oxygen saturation (SpO_2) levels show as normal despite breathing difficulties?
28. Why can SpO2 levels drop without warning and at what point should medical help be sought?
**Neurological**
**29. Overarching theme: Is cognitive impairment temporary or permanent? (e.g. inability to concentrate, to absorb or retain information, to spell or find the correct word, etc.)**

Houchen-Wolloff L, *et al. Thorax* 2022; 77:717–720. doi: 10.1136/thoraxjnl-2021-218582

30.	What is the cause of the autonomic nervous system dysfunction reported by many patients [PoTS, chronic fight or flight, mast cell activation syndrome (MCAS], and what can be done to help with this?
31.	To what extent are mental health symptoms (depression, anxiety) the result of physiological effects (e.g. inflammation, the chronic issues with their sympathetic nervous system mentioned above)?
32.	Will there be studies into the role of the gut brain axis?
**Social Sciences**	
33.	What can be done to help people back to work?
34.	What can be done to alleviate the stigma associated with Long Covid?

Houchen-Wolloff L, *et al. Thorax* 2022; 77:717–720. doi: 10.1136/thoraxjnl-2021-218582

Table S2 Full list of questions from Working Groups. Contains results for process stage 1.

**Lung Fibrosis Working Group**
1. How common is fibrotic or interstitial lung disease in Long COVID, and who gets it and why?
2. How can we best identify and characterise Long COVID Interstitial Lung Disease (LCILD), including the more subtle cases of LCILD, that may be missed on standard imaging, and understand how these contribute to the symptoms of Long COVID?
3. What happens to patients with LCILD; is LCILD time-limited inflammation and reversible, or does it develop into progressive fibrosis, and what are the risk factors and biomarkers for disease progression?
4. Can we understand the underlying pathology of LCILD: the role of epithelial cells, vascular cells and immune response and so identify prognostic and theragnostic biomarkers that will guide joint decision making?
5. By understanding pathomechanisms can we determine which conventional and novel therapeutic approaches may be useful to prevent/ mitigate the development of LCILD?
**Pulmonary and Systemic Vasculature**
1. What are the physical impacts for patients of pulmonary vascular disease in the convalescent phase of COVID-19?
2. How frequent are pulmonary vascular complications and do we need to screen patients for them?
3. What are the underlying biological reasons the pulmonary vessels are prominently affected in COVID-19?
4. If a significant amount of patients are suffering from pulmonary vascular complications, what treatments should we focus on?
5. What are the long-term consequences of the pulmonary vascular pathology in COVID-19 and in particular are patients susceptible to further blood clots?
**Intensive Care (ICU) Working Group**
1. What are the symptoms and patterns of recovery at 6 months in those patients ventilated on ICU with severe COVID?
2. What is the role of ongoing inflammation in the recovery of ICU patients with COVID?
3. Can we use data from (1) and (2) to inform future clinical trials of therapies to improve the quality of life of patients recovering from COVID critical illness?
**Airways Disease Working Group**
1. Exploring how the admission ISARIC 4C prognostic score (a risk stratification score that predicts in-hospital mortality for hospitalised COVID-19 patients) correlate with post discharge morbidity at 3 and 12 months in patients with pre-existing airways disease compared to those with none.
2. Determining if the 3- and 6-month post-COVID-19 fatigue, sarcopenia, anxiety and depression scores are worse in patients with pre-existing airway diseases as compared to those with no airways disease? Is this significant when adjusted for severity of in-patient disease?
3. Investigating if patients with pre-existing airway diseases who were treated for severe COVID-19 are at higher risk of future cardiovascular complications e.g. myocardial infarction, stroke at 6 and 12 months in comparison to those with no pre-existing airway disease.
4. Understanding the predictors of hospital re-admission post-COVID-19 in patients with pre-existing airways.
5. Developing and validating tools for remote monitoring, and to help people self-monitor symptoms, especially in patients with pre-existing airway diseases.
6. Determining the incidence and risk factors of new onset symptomatic obstructive airways

Houchen-Wolloff L, *et al. Thorax* 2022; 77:717–720. doi: 10.1136/thoraxjnl-2021-218582

disease in COVID-19 survivors, defined clinically and/or using objective diagnostic tests, e.g. spirometry and computerised tomography (CT) imaging at 3 & 12 months.
7. Assessing if current smokers fair worse in terms of recovery in patients with Chronic Obstructive Pulmonary Disease (COPD) who have had severe COVID-19 pneumonia.
8. Comparing the clinical and cost-effectiveness of exercise and education-based rehabilitation to improve health status compared with standard care in COVID-19 survivors with pre-existing airways diseases.
9. Exploring the benefit of progressive/bespoke exercise rehabilitation in people with airways disease and persistent symptoms or physical limitation delivered live or virtually following discharge after COVID-19 infection.
10. Assessing whether the outcomes of acute hospitalization with COVID-19 in patients with pre-existing airways disease on pre-COVID-19 long-term anticoagulants are better than those who were not on long-term anticoagulants (e.g. using in patient length of stay, discharge rates, long COVID-19 markers at 3, 6 and 12 months).
**Imaging Working Group**
1. What is prevalence of persistent multi-organs injury among COVID-19 survivors? [Magnetic resonance imaging (MRI), imaging biomarkers, blood tests, lung function test, standard clinical radiology].
2. What are the determinants/risk factors of persistent multi-organs injury? – Role of age, sex, comorbidities, body mass index (BMI), race, smoking, metabolic, socioeconomic background, genetics, immune mediators, preexisting disease, disease severity acutely, medical therapy in hospital, days since symptom onset/
3. Is there an association between multi-organs MRI injury and Long Covid symptoms?
4. What are the long-term outcomes/ prognostic implications of multi-organs injury on MRI? – end organ damage, hospitalisation, death, ongoing symptoms e.g. fatigue, chest pain, permanent disability.
5. Is there an association between MRI markers of organ injury and non-MRI quantitative and qualitative measurements of end organ injury at admission and follow-up e.g. Renal stages and glomerular filtration rate (GFR: a measure of kidney function), brain abnormalities and Montreal Cognitive Assessment (MoCA) scores, cardiac T1 on MRI and brain natriuretic peptide (BNP) and Troponin, lung T2 and computed tomography (CT) chest, electrocardiogram (ECG) and cardiac abnormalities on MRI.
6. Is there an association between antibody response or viral polymerase chain reaction (PCR) titres in the acute setting and multi-organs injury on follow up MRI?
7. What is the impact of therapy (e.g. dexamethasone) on multi-organs health in COVID-19 survivors?
8. Is there an association between psoas muscle T1 (on MRI) and measures of muscle strength, fatigue and other measures of sarcopenia?
9. What is the impact of multi-organs injury on mental health and vice versa (i.e., are patients with anxiety/depression before COVID more likely to have multi-organs injury)?
10. Is there an association between detailed immunology markers and multi-organs MRI abnormalities?
11. Are there differences in prevalence of multi-organ MRI abnormalities in hospitalised and non-hospitalised patients (from long-COVID multi-organ imaging studies)?
12. Are there differences in multi-organ tissue abnormalities between ICU and non-ICU patients?
13. Does multi-organ injury predispose to long-term emotional and physical disability in survivors?
14. What are the effects of multi-organ injury on exercise capacity and quality of life measures?
15. Are there any associations between multi-organ injury and the ’omics (proteomics, genomics)?

Houchen-Wolloff L, *et al. Thorax* 2022; 77:717–720. doi: 10.1136/thoraxjnl-2021-218582

16. Is there an association between insulin resistance among COVID-19 survivors and extent of multi-organ injury?
**Rehabilitation working group**
1. What is the value of a multi-disciplinary recovery programme for the post-COVID population compared to usual care. Outcomes would include but not limited to - health related quality of life, fatigue, activity, cognition.
2. What is the role of *Your COVID Recovery* digital programme in supporting recovery compared to best usual care? Outcomes would include but not limited to - health related quality of life, fatigue, activity, cognition.
3. 'Boom and Bust' physical activity pattern in COVID Recovery: to investigate the pattern of this cycle to identify its timeframe and association with health indicators and subjective markers of fatigue and wellbeing.
4. Can we identify the treatable traits of individuals who are likely to need a rehabilitation/ recovery programme?
**Sarcopenia Working Group**
The sarcopenia working group has a core question of whether loss of muscle mass, strength and function (sarcopenia) is one of the factors contributing to Long Covid, especially the poor quality of life and fatigue. Linked to this we have research questions that stem from this:
1. Does the infiltration of immune cells into muscle associate with sarcopenia? Are there specific immune cells that associate with the presence or absence of sarcopenia, i.e. a poor or good recovery after Covid? If so, we could target specific immune cells to improve outcomes.
2. What is the metabolic and functional status in patients post Covid following a severe admission? This involves novel dynamic MRI - studying the patient whilst they are exercising to assess brain, cardiac and muscle function to give a holistic view of the patient. Additionally, there is detailed metabolic assessment of the patient, muscle biopsies and muscle function.
3. Is the immune system and muscle more aged biologically, giving an aged and frail phenotype? If so, then targeting ageing processes with drugs such as metformin could reverse this ageing effect.
**Fatigue working Group**
Although fatigue is described by many long-COVID patients and recognised by health care professionals caring for these patients little is known about the patient perspective. We propose a qualitative study is to answer the following questions:
1. How do patients with post-COVID fatigue characterise their symptoms?
2. How do they describe their experience of fatigue and what burdens does it place on daily living?
3. How have they managed their symptoms? What resources, if any, have they used?
**Brain Working Group (Includes Mental Health & Neurology)**
1. Who - in terms of psychosocial factors - becomes ill with Long Covid?
2. What causes Long Covid? (Persistent virus, aberrant immune response/inflammation, autoimmunity, other)
3. To what extent are mental health symptoms [and corresponding disorders of post-traumatic stress disorder (PTSD), depression, and anxiety) present in the first 1000 patients experiencing COVID-19?
4. How common is cognitive impairment (e.g. inability to concentrate, to absorb or retain information, to spell or find the correct word, etc.) Who gets it and is cognitive impairment temporary or permanent?

Houchen-Wolloff L, *et al. Thorax* 2022; 77:717–720. doi: 10.1136/thoraxjnl-2021-218582

5. What is the prevalence and severity of hearing loss, tinnitus, and imbalance?
6. What is the underlying reason for ongoing breathing problems? Do these relate to the lungs, heart or nervous system?
7. Are there specific inflammatory protein profiles/immune cell phenotypes in post-COVID patients who develop depression/anxiety, cognitive dysfunction and fatigue symptoms?
8. What can be done to aid recovery including helping people get back to work? Does integrating physical rehabilitation with a psychological approach help?
**Cardiovascular Working Group**
Qualitative and quantitative biomarkers for outcome prediction
1. Is there a signature/s of biomarkers that, in conjunction with clinical history and PHOSP imaging assessments, would allow you to predict cardiovascular (CV) outcomes of patients who have been hospitalised with COVID-19? How do signature/s of gold-standard clinical biomarkers (quantitative) compare to those identified via Olink Explore 1536 or specific panels (qualitative)?
2. Does quantification of vascular inflammation from CT pulmonary angiogram (CTPA)/ chest CT scans of hospitalised COVID-19 patients allow to predict risk of adverse outcomes?
Signs of CV disease in clinical assessment of hospitalised COVID-19 patients
3. Are there specific electrocardiogram (ECG) patterns associated with myocardial inflammation and/or compromised pulmonary circulation?
Mechanisms of disease
4. Is the intensity of cytokine release in response to SARS-CoV-2 infection determined by specific major histocompatibility complex- human leukocyte antigen (MHC-HLA) and killer-cell immunoglobulin-like receptor (KIR) genotypes? If so, would these genotypes be predictors of increased risk of relapse, readmission or mortality?
5. Do endothelial cell-derived microparticles (ECMPs) increase as a result of COVID-19? Could ECMPs function as biomarkers and/or predictors of cardiovascular dysfunction as a result of Covid-19? Can ECMPs facilitate transport of SARS-CoV-2 virus?
CVD & Functional outcomes
6. How do the different forms of pre-existing cardiovascular disease (eg HF, CAD, PAD) relate to long-term functional recovery and quality of life in PHOSP COVID participants? Are these independent of other risk factors e.g. age, DM, renal impairment, previous PAD or other vascular disease(s)?
**Metabolic Working Group**
1. Do people with (different types of) diabetes hospitalised with COVID-19 have higher rates of short-/medium-/long-term complications than those without (those types of) diabetes?
2. What mechanisms explain any increased rates in people with diabetes?
3. Within people with (different types of) diabetes and COVID-19 what factors (baseline demographics/ comorbidities/ metabolic control/ biomarkers) predict a 'full' recovery at 28 days/ 3 months/ 12 months, controlling for COVID-19 disease severity?
4. Does the presence of (any type of) diabetes modify the efficacy of any medical (e.g. steroids), psychological, or technological intervention known to have a beneficial effect on biomedical, psychological, or social outcomes of COVID-19?
5. What is the long-term impact of COVID-19 on metabolic and risk factor control in people with diabetes (glycaemia, blood pressure (BP), weight, lipids, etc.)?
6. Do people with diabetes experience greater mental health difficulties than other groups due to particular challenges in maintaining lifestyles, social isolation and keeping glucose levels at target?

**Renal Working Group**
1. What is the proportion of people with new Chronic Kidney Disease (CKD) post COVID-19 disease?
2. What is the proportion of people with worsened CKD post COVID-19 disease?
3. What are the risk factors for points 1 and 2 - these may include severity of existing CKD, severity of COVID-19 disease, diabetes, hypertension, known vascular disease, proteinuria?
4. Is there a discrepancy between glomerular filtration rate (GFR: a measure of kidney function) estimated by cystatin C and that estimated by conventional methods? This would address how COVID-related sarcopenia (loss of muscle) might change our thresholds for diagnosing COVID-19 related kidney injury?
5. Does COVID-19 infection affect patients on the kidney transplant waiting list - what proportion have been suspended from the wait list following COVID-19 disease?
6. Are there biomarkers for long term kidney damage (e.g. in the link studies) and how do they correlate with radiological findings e.g. C-MORE.
7. How is the functional status of dialysis patients (and those with CKD stage 4) affected by COVID-19 disease?
8. Can we integrate with other working groups, particularly "fatigue and sarcopenia" on functional status assessment and can the Kidney Beam Intervention improve functional status (e.g. exercise tolerance, fatigue) in renal patients who have had COVID-19 disease?
9. What is the impact of CKD and Renal Replacement Therapy on Covid immunity and vaccine responses?
**Immunology Working Group**
1. How long do immune aberrations remain in patients with prior COVID-19?
2. Which immune aberrations are associated with ongoing chest X-ray alterations?
3. Can the low COX-2 in monocytes be reverted by existing therapeutics, and if so which ones?
4. How long does T and B cell immunity last in post-COVID-19 patients and does this link with the original disease severity?
5. Does post-COVID immunity mean that vaccination is not a priority for this group?
6. Can post-COVID-19 patients mount effective anti-vaccine responses?
7. How do aberrant monocytes post-COVID-19 alter the induction of anti-viral/ vaccine T and B cells?

Houchen-Wolloff L, *et al. Thorax* 2022; 77:717–720. doi: 10.1136/thoraxjnl-2021-218582

Table S3. List of 24 questions sent to survey, following synthesis of questions according to overarching theme, undertaken by PHOSP-COVID Core Management Group and rephrasing from people with Long Covid. Results of process stage 2.

1.	What conditions such as autoimmune disorders, PoTS, mast cell activation (histamine intolerance, allergies) or damage to individual organs (such as lungs, heart and kidneys) may be caused by Long Covid? [expansion of question, specific to new conditions]
2.	What are the risk factors (e.g. age, sex, ethnicity, socioeconomic factors) for Long Covid? [expansion of question]
3.	What are the risk factors during the initial Covid-19 infection (such as severity) that might predict organ damage (lungs, heart, kidneys)? [language made accessible]
4.	What imaging techniques or scans (e.g. MRI, CT) may be able to detect and predict the development of organ problems or wider systemic issues? [revision of question]
5.	What blood or other laboratory tests may be able to detect and predict the development of organ problems or wider systemic issues? [revision of question]
6.	What are the physical effects of Long Covid during the first year, post infection? [overarching question which links another question]
7.	What is the risk of future adverse health events (e.g. stroke, heart attack)? [new question from patients]
8.	Why do symptoms fluctuate and new symptoms develop? [overarching question]
9.	What can data at 3, 6 and 12 months tell us about the long-term trajectory of illness? [revision of question]
10.	What are the underlying mechanisms of Long Covid (e.g. autoimmunity, viral persistence, inflammation) that drive symptoms and/or organ impairment? [combination of 2 questions and language made accessible]
11.	What is the impact of treatment(s) given after discharge from hospital on their recovery? [language made accessible]
12.	What is the impact of treatment(s) during the acute (initial) stage of COVID-19 on recovery? [language made accessible]
13.	What are the biological mechanisms causing mental ill health, for example brain inflammation, adrenal response, gut health)? [new question from patients]
14.	What can be done to support mental wellbeing during recovery, e.g. trauma counselling? [new question from patients]
15.	What medications, dietary changes, supplements, rehab and therapies aid recovery? [new question from patients]
16.	What are the factors that predict a good/poor response to exercise, to inform rehabilitation? [expansion of 1 question]
17.	What is the cause of worsening or relapse of symptoms after physical or mental activity during recovery? [new question from patients]
18.	What happens to the immune system throughout patients' recovery from COVID-19? [3 questions combined and language made accessible
19.	Where relevant, what is the impact of body weight in those recuperating from Covid-19 on recovery speed and long-term health? [question had been overlooked]
20.	What is the impact of a pre-existing long-term condition on recovery? [revised and language made accessible]
21.	What are the best ways to organise services for patients recovering from COVID-19? [2 questions combined and language made accessible]
22.	How does COVID-19 and its aftereffects cause the body to age more quickly? If so, what can be done to slow this process to help people improve their daily activities/function? [language made accessible]

Houchen-Wolloff L, *et al. Thorax* 2022; 77:717–720. doi: 10.1136/thoraxjnl-2021-218582

23.	What can be done to help people back to work? [kept, no change]
24.	What are the problems within the muscles [causing early or delayed high levels of lactic acid release] associated with symptoms limiting activity/function/exercise? If so, what can be done to help? [kept, no change]

Figure S1: Map of PHOSP Working Groups and Patient Charities

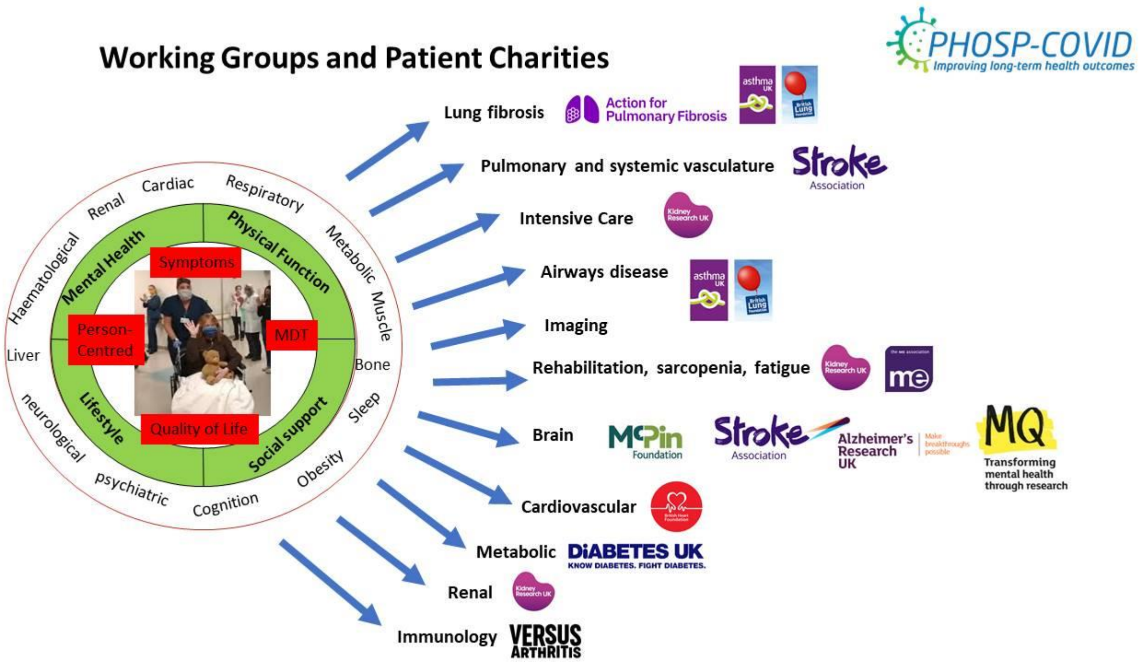

Houchen-Wolloff L, *et al. Thorax* 2022; 77:717–720. doi: 10.1136/thoraxjnl-2021-218582

Figure S2: Summary of the Prioritisation Workshop Process

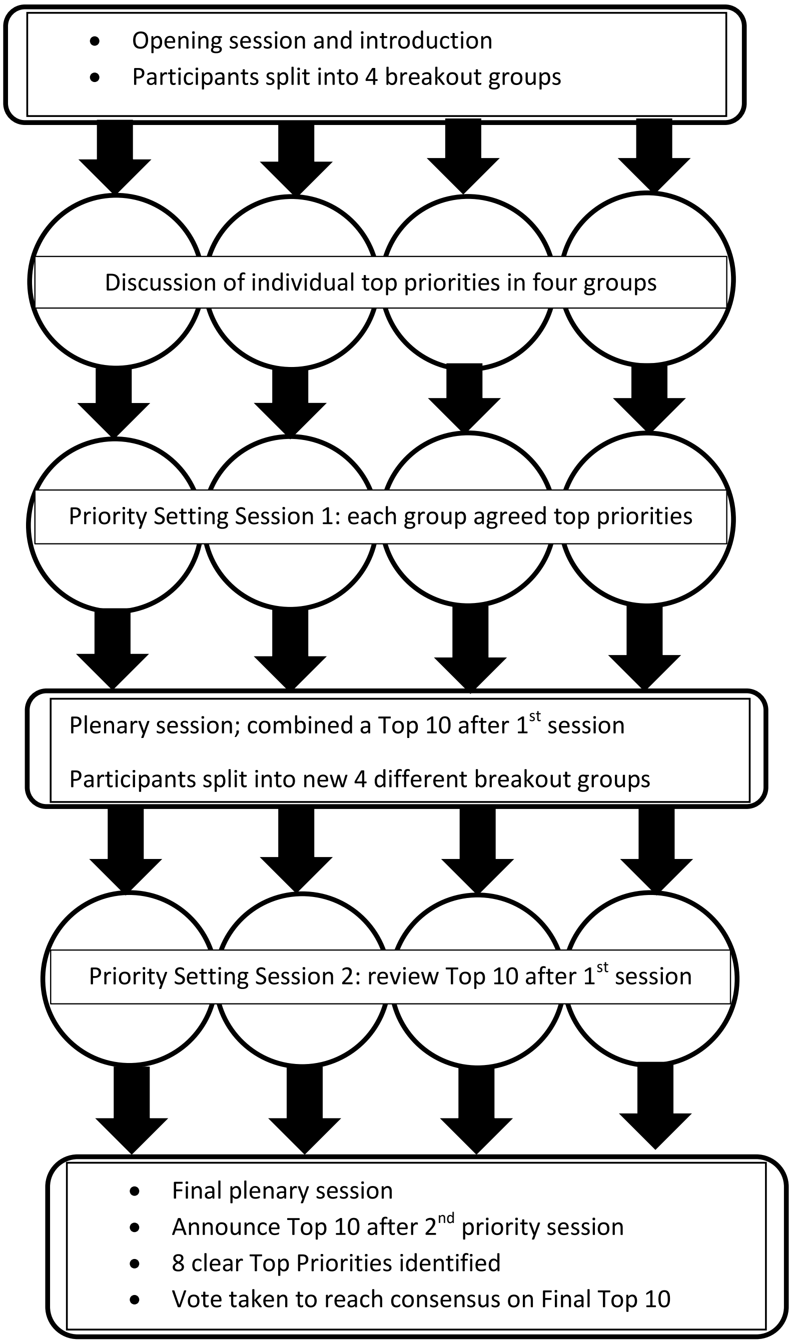

Houchen-Wolloff L, *et al. Thorax* 2022; 77:717–720. doi: 10.1136/thoraxjnl-2021-218582
